# DYNamic Assessment of Multi‐Organ level dysfunction in patients recovering from COVID‐19: DYNAMO COVID‐19

**DOI:** 10.1113/EP091590

**Published:** 2024-06-24

**Authors:** Ayushman Gupta, Rosemary Nicholas, Jordan J. McGing, Aline V. Nixon, Joanne E. Mallinson, Tricia M. McKeever, Christopher R. Bradley, Mathew Piasecki, Eleanor F. Cox, James Bonnington, Janet M. Lord, Christopher E. Brightling, Rachael A. Evans, Ian P. Hall, Susan T. Francis, Paul L. Greenhaff, Charlotte E. Bolton

**Affiliations:** ^1^ NIHR Nottingham Biomedical Research Centre Nottingham UK; ^2^ Centre for Respiratory Research, Translational Medical Sciences, School of Medicine University of Nottingham Nottingham UK; ^3^ Nottingham University Hospitals NHS Trust Nottingham UK; ^4^ Sir Peter Mansfield Imaging Centre, School of Physics & Astronomy University of Nottingham Nottingham UK; ^5^ David Greenfield Human Physiology Unit, School of Life Sciences University of Nottingham Nottingham UK; ^6^ MRC‐Versus Arthritis Centre for Musculoskeletal Ageing Research University of Nottingham Nottingham UK; ^7^ MRC‐Versus Arthritis Centre for Musculoskeletal Ageing Research University of Birmingham Birmingham UK; ^8^ NIHR Birmingham Biomedical Research Centre University of Birmingham Birmingham UK; ^9^ NIHR Leicester Biomedical Research Centre University of Leicester Leicester UK

**Keywords:** COVID‐19, pathophysiology, post‐acute COVID‐19 syndrome, recovery

## Abstract

We evaluated the impacts of COVID‐19 on multi‐organ and metabolic function in patients following severe hospitalised infection compared to controls. Patients (*n *= 21) without previous diabetes, cardiovascular or cerebrovascular disease were recruited 5–7 months post‐discharge alongside controls (*n* = 10) with similar age, sex and body mass. Perceived fatigue was estimated (Fatigue Severity Scale) and the following were conducted: oral glucose tolerance (OGTT) alongside whole‐body fuel oxidation, validated magnetic resonance imaging and spectroscopy during resting and supine controlled exercise, dual‐energy X‐ray absorptiometry, short physical performance battery (SPPB), intra‐muscular electromyography, quadriceps strength and fatigability, and daily step‐count. There was a greater insulin response (incremental area under the curve, median (inter‐quartile range)) during the OGTT in patients [18,289 (12,497–27,448) mIU/min/L] versus controls [8655 (7948–11,040) mIU/min/L], *P *< 0.001. Blood glucose response and fasting and post‐prandial fuel oxidation rates were not different. This greater insulin resistance was not explained by differences in systemic inflammation or whole‐body/regional adiposity, but step‐count (*P* = 0.07) and SPPB scores (*P* = 0.004) were lower in patients. Liver volume was 28% greater in patients than controls, and fat fraction adjusted liver *T*_1_, a measure of inflammation, was raised in patients. Patients displayed greater perceived fatigue scores, though leg muscle volume, strength, force‐loss, motor unit properties and post‐exercise muscle phosphocreatine resynthesis were comparable. Further, cardiac and cerebral architecture and function (at rest and on exercise) were not different. In this cross‐sectional study, individuals without known previous morbidity who survived severe COVID‐19 exhibited greater insulin resistance, pointing to a need for physical function intervention in recovery.

## Abstract (highlights)

- **What is the central question of the study?**
  What are the post‐COVID‐19 symptoms and associated metabolic and physiological sequelae in patients who contracted acute severe infection compared to healthy control volunteers?
- **What is the main finding and its importance?**
  Patients 5–7 months after hospital discharge for acute severe COVID‐19 compared to healthy control volunteers had (i) an increased insulin response to an oral glucose challenge, without demonstrating different whole‐body fuel oxidation rates, and (ii) greater perception of fatigue and worse functional mobility, though no abnormalities in muscle, heart or brain structure and function were identified. This provides novel targets for rehabilitation strategies in individuals recovering after severe COVID‐19.

## 1 INTRODUCTION

With the huge surge in cases of COVID‐19 since early 2020, the initial management priority was to reduce the spread and severity of the acute infection (Faden et al., 2020; NICE, 2021). The majority of the population are now surviving the acute illness, but the longer‐term sequelae of COVID‐19 are manifesting as a major cause of morbidity for millions (Han et al., 2022). The focus is now on addressing the long‐term consequences, which can limit activities of daily living, work productivity and lifestyle, even in people with no prior medical illness or morbidity (Shah et al., 2021). A wealth of literature highlights the persistent and limiting symptoms in patients following SARS‐Cov‐2 infection, including greater fatigue perception, myalgia, ‘brain fog’ and breathlessness (Evans et al., 2021). The large UK multi‐centre Post‐HOSPitalisation COVID‐19 (PHOSP‐COVID) multicentre study reported that 52% of individuals felt that they had not made a full recovery 6 months after resolution of acute severe COVID‐19 (Evans et al., 2021), with a similar number not recovering at 1 year (Evans et al., 2022).

During the acute phase of the infection, a spectrum of organ damage and dysfunction can occur, including rhabdomyolosis, myocarditis, pneumonitis and cerebral neuropathy (Zaim et al., 2020). However, little is known of the extent to which tissue damage persists and contributes to long‐term symptoms. Publications have reported multi‐organ abnormalities in the short to medium term post‐COVID‐19 compared to controls (Dennis et al., 2021; Raman et al., 2021). However, such studies have assessed patients in the resting state, and have not included stressors to physiological homoeostasis such as low‐intensity exercise, which reflects everyday circumstances where symptoms are usually reported. Further, contemporaneous evaluation of metabolic homoeostasis resulting from feeding challenges is lacking. This is despite an increased frequency of new‐onset type 2 diabetes (T2DM) and glucose intolerance observed from 6 months post SARS‐Cov‐2 infection (Montefusco et al., 2021; Xie & Al‐Aly, 2022) for which mechanistic insight is missing. There is a need to identify physiological and metabolic maladaptation contributing to persisting symptoms, particularly perceived fatigue in people recovering from COVID‐19.

Here, we undertook detailed metabolic and physiological phenotyping of patients who survived severe SARS‐CoV‐2 infection as well as healthy control volunteers, which included dynamic measurements during acute dietary and exercise stress to best elucidate pathophysiology. This focuses on two of the top 10 research questions in the joint patient and clinician Priority Setting exercise for people recovering from COVID‐19: (i) the mechanisms that drive symptoms ± organ impairment and (ii) what the problems within the muscle are. To further examine mechanisms contributing to fatigue, we conducted a sub‐group analysis between patients with and without perception of fatigue.

## 2 METHODS

### 2.1 Ethical approval

This study was registered at ClinicalTrials.gov (NCT05060497) and approved by the West London Research Ethics Committee, UK (20/HRA/3612). The experiments were conducted in humans and all participants gave written informed consent. The study conformed to the standards set by the *Declaration of Helsinki*.

### 2.2 Study population

Adults (≥18 years old), with laboratory‐confirmed COVID‐19 infection that required hospital admission, classified as a severe admission, between March 2020 and July 2021, were recruited within 5–7 months of discharge from acute hospital Trusts, East Midlands, UK. Healthy controls, with no previous evidence of COVID‐19 infection of a similar age, sex, body mass index (BMI) and ethnicity as patients were also recruited. The project was advertised through flyers, social media platforms, departmental mailing lists and website domains for potential healthy participants to contact the team if they were interested. Exclusion criteria were absolute contraindications for MR scan, pre‐COVID diagnosis of chronic respiratory, renal, cardiovascular or cerebrovascular disease or diabetes, BMI < 20 kg/m^2^, pregnancy, those requiring long‐term oxygen therapy or non‐invasive ventilation and the inability to carry out a sustained period of supine stepping exercise.

### 2.3 Study procedures

**FIGURE 1:**
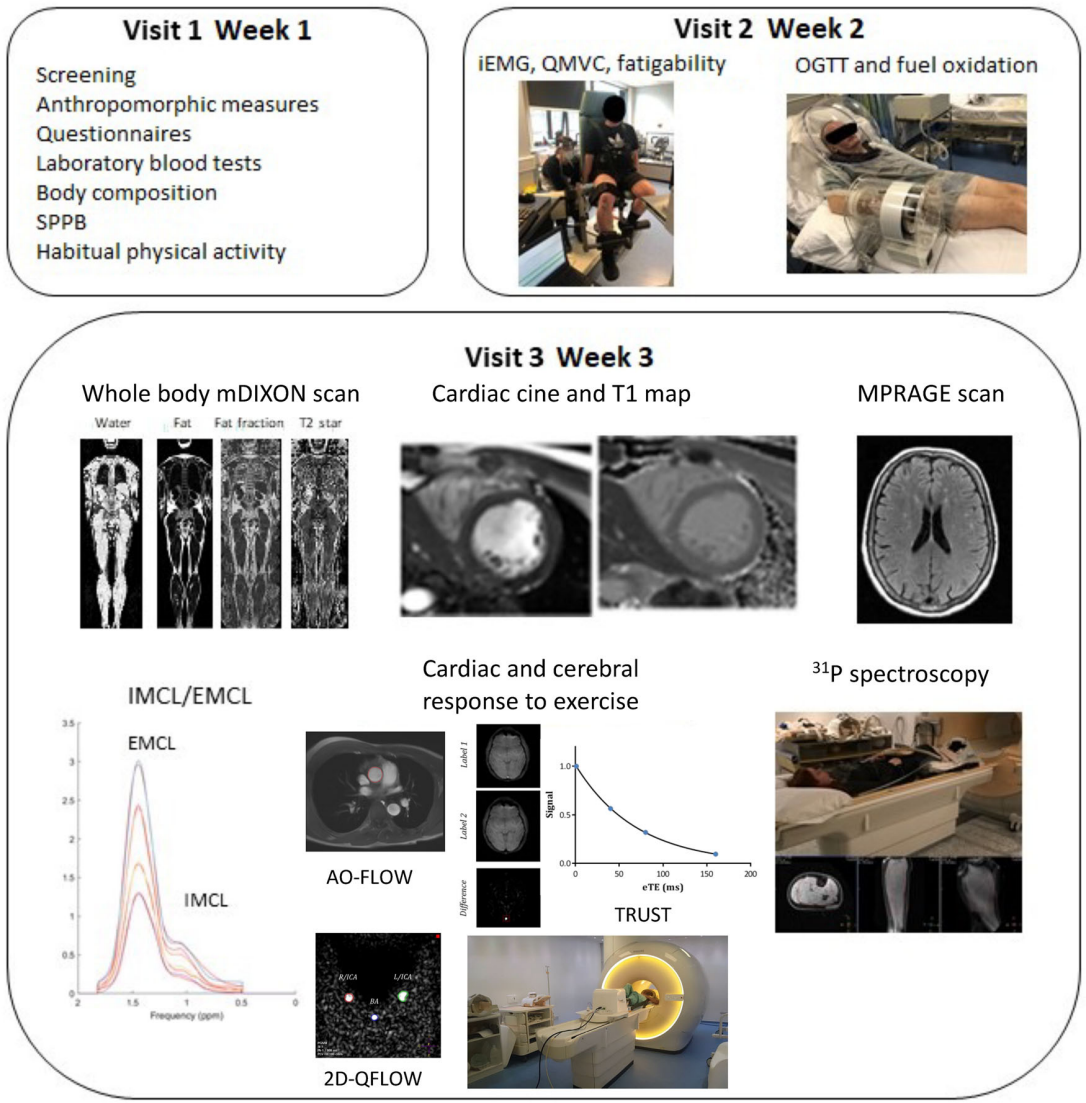
Experimental procedures conducted during the three study visits. Visit 1 included screening of participants followed by measures of whole‐body composition, symptomatology, blood tests, and physical function. Visit 2 comprised sampling of arterialised venous whole blood glucose and serum insulin concentrations in conjunction with whole‐body fuel oxidation rates during an oral glucose tolerance test. Quadriceps isometric strength and isokinetic work output (20 fatiguing maximal knee extensions) were quantified using Cybex dynamometry. Visit 3 comprised MR imaging and spectroscopy protocols involving whole body mDIXON: measures of muscle mass and fat; MPRAGE: brain architecture; short axis cine: resting cardiac function; cardiac *T*_1_ mapping: myocardial inflammation; ^1^H MR spectroscopy: intra‐ and extra‐myocellular lipid concentration. Measures in response to exercise involved 2D‐QFLOW: cerebral blood flow; TRUST: oxygenation; AO‐FLOW: cardiac output and ^31^P MR spectroscopy: muscle phosphocreatine resynthesis. EMCL, extra‐myocellular lipids; iEMG, intra‐muscular electromyography; IMCL, intra‐myocellular lipids; OGTT, oral glucose tolerance test; QMVC, quadriceps maximal voluntary isometric contraction; SPPB, short physical performance battery.

The methods are outlined in Figure 1 and comprised three study visits within a 3‐week window. Height and body weight were measured to calculate BMI and body surface area (BSA) (Mosteller, 1987), with BMI used as an initial screening surrogate to gauge patients and controls of similar body fat mass. Body composition was formally assessed using dual‐energy X‐ray absorptiometry (DEXA) scan (Lunar Prodigy Advance, GE Healthcare, Chicago, Illinois) (Laskey, 1996).

The Fatigue Severity Score (FSS) (Krupp et al., 1989) with perceived fatigue classed as 36 or more (Herlofson & Larsen, 2002), dyspnoea‐12 (Yorke et al., 2010), Montreal Cognitive Assessment (MoCA) (Nasreddine et al., 2005) tool and patient health questionnaire‐9 (PHQ‐9) (Martin et al., 2006) were completed.

Venous blood was analysed for creatine kinase (CK), full blood count, glycosylated haemoglobin (HbA1C), liver function tests (LFTs), brain natriuretic peptide (BNP), troponin and ferritin in the United Kingdom Accreditation Service (UKAS) laboratories at Nottingham University Hospitals NHS Trust. Aliquots of serum were also stored at −80°C following centrifuging and analysed for C‐reactive protein (CRP), tumour necrosis factor‐α (TNFα) and interleukin‐6 (IL‐6) concentrations by enzyme‐linked immunosorbent assay (ELISA) on batched samples (Quantikine ELISA kit, R&D Systems, Minneapolis, MN, USA).

A physical activity armband monitor (Sensewear®, APC Cardiovascular Ltd, Northwich, UK) was worn on the upper arm for a week during waking hours, from which the average daily step count was determined for each volunteer.

Quadriceps muscle isometric strength (quadriceps maximal voluntary contraction, QMVC) was determined through three maximal isometric knee extensions, with participants’ dominant leg fixed at a 60° angle, interspersed with 60 s rest between efforts, using a Cybex dynamometer (HUMAC2009®/NORMTM, Phoenix Healthcare, Nottingham, UK). The best of the three measurements was taken. Following a 10‐min recovery period, muscle work output during 20 repeated maximal voluntary knee extensions was also assessed for muscle fatiguability, at a constant angular velocity of 90°/s. The magnitude of the decline in work output (fatigue) was compared between groups.

A short physical performance battery (SPPB) test quantified physical function (Guralnik et al., 1994). Motor unit potential (MUP) properties within the vastus lateralis (VL) during isometric contraction at 25% QMVC were assessed using intra‐muscular electromyography (iEMG) (Guo et al., 2022).

An oral glucose tolerance test (OGTT) and concurrent indirect calorimetry were performed to determine whole‐body glucose disposal, insulinaemia and fuel oxidation rates (Schadewaldt et al., 2013; Shur et al., 2022). Participants were instructed to fast overnight, avoid any strenuous exercise in the 48‐h period prior to the laboratory visit and also refrain from caffeine and alcohol consumption for 24 h prior. During the study visit, a retrograde cannula was inserted into a dorsal hand vein and then placed in a hot warming unit (55°C), which allowed collection of arterialised venous blood for glucose and insulin sampling (Gallen & Macdonald, 1990). For whole‐blood glucose, samples were drawn in a fasting state and then concurrently every 10 min following a 75 g oral glucose challenge for the initial 2 h and every 15 min for the final hour. An additional 1 mL of arterialised venous blood was obtained in a fasting state and then every 20 min following the challenge for the first 2 h and every 30 min for the final hour to assess serum insulin concentration. A glucose analyser machine (YSI 2300 STAT Plus, Yellow Springs Inc., Yellow Springs, OH, USA) was used to quantify real‐time blood glucose concentrations, whilst the other serum samples were stored at −80°C for analysis of insulin concentration at a later stage, using the ELISA method on batched samples (Quantikine ELISA kit, R&D Systems). Whole‐body insulin sensitivity was estimated using the Matsuda Index (Matsuda & DeFronzo, 1999). Incremental area under the curve (iAUC) for insulin and glucose concentrations measured during the OGTT was calculated using the trapezoid method (Arvidsson‐Lenner et al., 2004).

The indirect calorimetry involved measuring steady‐state resting oxygen (O_2_) consumption and carbon dioxide (CO_2_) production using a ventilated hood system (GEMNutrition Ltd, Daresbury, UK) before and during the OGTT to estimate fat and carbohydrate oxidation rates (Frayn, 1991).

### 2.4 Magnetic resonance imaging and spectroscopy

Volunteers underwent proton (^1^H) magnetic resonance imaging (MRI) and spectroscopy (MRS) on a 3T Philips Ingenia scanner (Philips Medical Systems, Best, Netherlands) and phosphorus (^31^P) MRS on a 3T Philips Achieva scanner in a supine position to study organs in the resting state and in response to exercise.

MRI sequences collected at rest included whole body mDIXON for calf and thigh muscle volume (adjusted for BSA) and intra‐muscular fat fraction (FF) for adiposity, as well as measures of liver volume, adiposity and *T*_2_* representing iron content. Calf and thigh muscle volume and the intra‐muscular FF measures were extracted using a semi‐automated MATLAB (MathWorks, Natick, MA, USA) script. Regions of interest (ROI) were drawn manually around the liver using Medical Image Processing, Analysis and Visualization (MIPAV) software to estimate liver volume, liver FF and *T*_2_^*^. Liver *T*_1_ corrected for liver FF was measured using a shortened Modified Look‐Locker Inversion (shMOLLI) scheme. Intra‐myocellular (IMCL) and extra‐myocellular (EMCL) lipid content in the VL were acquired using ^1^H MRS with MATLAB analysis. An MPRAGE brain scan was conducted from which grey matter (GM), white matter (WM) and cerebrospinal fluid (CSF) volume, as well as cortical thickness were determined (Computational Anatomy Toolbox 12 (CAT12) software, Wellcome Department of Cognitive Neurology, UK). Cardiac shMOLLI assessed the longitudinal relaxation time (*T*_1_) of the myocardium and left ventricle (LV) short‐axis cine measured cardiac output indexed to BSA (iCO) at rest (Schulz‐Menger et al., 2020).

Phosphocreatine (PCr) resynthesis in the medial gastrocnemius muscle following ischaemic in‐bore plantar flexion exercise (Trispect, Ergospect GmBH, Austria) during occlusion was measured using ^31^P MRS to estimate in vivo muscle mitochondrial function (Kemp et al., 2015) (jMRUI software). Interleaved measures of iCO (using aortic flow), grey‐matter cerebral blood flow (gmCBF) (using 2D‐QFLOW, analysed using ViewForum software, Philips Medical Systems, Netherlands) and grey‐matter corrected cerebral metabolic rate of oxygen (gmCMRO) using T(2)‐relaxation‐under‐spin‐tagging (TRUST) (Lu & Ge, 2008) were performed at rest, during in‐bore steady‐state supine stepping exercise and during recovery. For the supine stepping, a standardised exercise protocol was employed using an MRI‐compatible cardiostepper ergometer (Ergospect GmBH, Innsbruck, Austria). This involved supine steady‐state isokinetic stepping exercise, at a cadence of 70 steps a minute at a workload equivalent to 60% of the maximum predicted heart rate (HR) (workload that achieved the target HR was identified from a sub‐maximal incremental supine exercise test conducted at study visit 1, and during recovery). A peripheral pulse (PPU) finger monitor was used to measure HR before and during in‐bore exercise.

### 2.5 Statistical analysis

Statistical analyses were conducted using Stata SE V.15 (StataCorp, College Station, TX, USA) and GraphPad Prism V.9 (GraphPad Software, Boston, MA, USA). Continuous variables were tested for normality and represented as either medians and inter‐quartile range (IQR) if non‐parametric or means and standard deviation (SD) if parametric. Categorical data were reported as frequency and percentages. Mann–Whitney and Wilcoxon tests were used to compare binary groups of continuous non‐parametric variables, whilst an unpaired two‐tailed Student's *t*‐test was performed for parametric data. Comparison between categorical variables was assessed through a chi‐squared test. Correlation between continuous variables was assessed through Pearson's correlation coefficient. For iEMG, a multi‐level mixed effects linear regression model was used to investigate motor unit parameters for patients versus controls. Multivariate regression was used to adjust for confounding variables, when comparing outcome variables between groups; multivariate regression comparing Matsuda Index between patients and controls was adjusted for BMI as a covariate and percentage change in gmCBF from rest to exercise between patients with and without perceived fatigue was adjusted for cardiac output indexed to BSA change with exercise. These covariates included were based on a priori knowledge. Mixed effects two‐way ANOVA enabled comparisons between groups across multiple time points, with Šidák's multiple comparison test providing adjusted *P*‐values. To identify factors leading to fatigue, the patient group was further delineated as those with and without perceived fatigue, according to FSS. Significance was accepted at *P* < 0.05.

The study sample size of 20 patients and 10 controls was a *priori* calculated to detect with 80% power based on literature utilising similar methodology and the most relevant patient groups for the following parameters: PCR recovery kinetics (Menon et al., 2021), fatiguability (Menon et al., 2012), QMVC (Latimer et al., 2022), cerebral blood flow (CBF) (Hale, 2018) and cardiac index (Breidthardt et al., 2015) with a 2:1 ratio, using similar methodology as this study and the most relevant patient groups.

## 3 RESULTS

**FIGURE 2:**
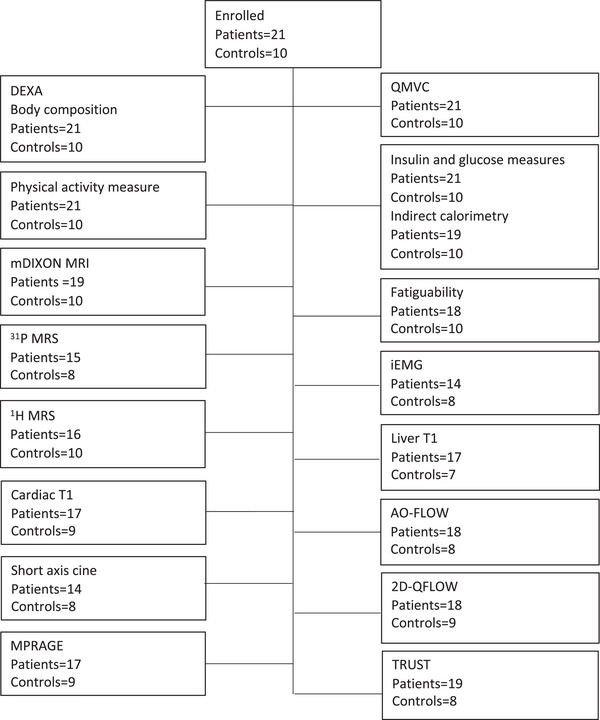
Sample size for successful completion of experimental assessments. 2D‐QFLOW, cerebral blood flow; AO‐FLOW, aortic flow; DEXA, dual‐energy X‐ray absorptiometry; iEMG, intra‐muscular electromyography; MPRAGE, magnetization‐prepared rapid gradient‐echo; MRI, magnetic resonance imaging; MRS, magnetic resonance spectroscopy; QMVC, quadriceps maximal voluntary contraction; TRUST, *T*_2_‐relaxation‐under‐spin‐tagging.

Thirty‐one participants (21 patients post‐COVID‐19 and 10 controls) were enrolled into the study, of whom 19 patients and 10 controls completed the MR visit (Figure 2). The demographic data for all participants enrolled into the study, those who completed the MRI visit and the patients stratified according to perception of fatigue are presented in Table 1. BMI was greater in patients compared to controls, though the DEXA‐derived whole body FF was comparable between groups. Missing data were due in part to subject tolerability, technical failure or data quality.

**TABLE 1:** Demographic data for (A) all participants enrolled into the study and those participants that completed the MR visit, and (B) sub‐group analysis of patients with and without heightened perception of fatigue.

A	Patients	Controls	*P*
**Enrolled into study**			
*n*	21	10	
Age (years)	54 (49–61)	58 (52–67)	0.6
Male/female (*n*)	15/6	7/3	0.9
Ethnicity – White Caucasian [*n* (%)]	19 (95%)	10 (100%)	0.3
BMI (kg/m^2^)	32.4 (5.7)	28.4 (3.4)	0.06
Whole‐body FF %	38.3 (6.8)	37.4 (9.6)	0.8
**Completed MRI visit**			
*n*	19	10	
Age (years)	54 (50–60)	58 (52–67)	0.5
Male/female (*n*)	14/5	7/3	0.8
Ethnicity – White Caucasian [*n* (%)]	18 (95%)	10 (100%)	0.5
BMI (kg/m^2^)	33.0 (5.3)	28.4 (3.4)	0.02
DEXA‐derived whole‐body FF %	39.1 (6.6)	37.4 (9.6)	0.6

B	Fatigued patients	Non‐fatigued patients	*P*
**Enrolled into study**			
*n*	12	9	
Age (years)	54 (48–59)	56 (50–61)	0.6
Male/female (*n*)	8/4	7/2	0.6
Ethnicity – White Caucasian [*n* (%)]	10 (83%)	9 (100%)	0.2
BMI (kg/m^2^)	33.7 (5.8)	30.6 (5.4)	0.2
DEXA‐derived whole‐body FF %	39.8 (6.5)	36.2 (7.1)	0.2

*Note*: The data are presented as means (standard deviation), *n* (%) or medians (inter‐quartile range). Abbreviations: BMI, body mass index; FF, fat fraction derived from dual‐energy X‐ray absorptiometry; MRI, magnetic resonance imaging; MRS, magnetic resonance spectroscopy.

Acutely, more than half of patients received invasive mechanical ventilation, three (14%) required non‐invasive ventilation (NIV), 2/3 were prescribed dexamethasone, and the average hospital length of stay was 34 days. About 2/3 experienced persisting breathlessness and a similar proportion reported fatigue 5–7 months following discharge from the hospital. This was substantiated by the patient‐reported outcome measures (PROMs) reporting greater perception of fatigue and breathlessness compared to controls (Table 2). There were no differences in serum inflammatory cytokines measured, cardiac biomarkers, LFTs and HbA1c between groups (Table 2).

**TABLE 2:** Patient‐reported outcome measures and blood‐based biomarkers obtained at visit 1 for patients and controls, and inpatient history and post‐COVID‐19 symptoms in patients.

	Patients (*n* = 21)	Controls (*n* = 10)	*P*
Past medical and medications			
Anxiety/depression [*n* (%)]	2 (10)	1 (10)	0.9
Hypertension [*n* (%)]	3 (14)	2 (20)	0.2
Antidepressants [*n* (%)]	4 (19)	3 (30)	0.7
Beta blockers [*n* (%)]	4 (19)	1 (10)	0.6
Maximum level of treatment during inpatient stay			
IMV [*n* (%)]	11 (52)	NA	NA
NIV [*n* (%)]	3 (14)	NA	NA
High flow oxygen [*n* (%)]	7 (33)	NA	NA
Proportion on dexamethasone during in‐patient stay [*n* (%)]	14 (67)	NA	NA
Hospital length of stay (days)	34 (31)	NA	NA
Post‐COVID‐19 patient‐reported symptoms at the time of study visit			
Dyspnoea [*n* (%)]	13 (62)	NA	NA
Fatigue [*n* (%)]	14 (67)	NA	NA
Brain fog [*n* (%)]	6 (29)	NA	NA
Cough [*n* (%)]	6 (29)	NA	NA
Myalgia [*n* (%)]	8 (38)	NA	NA
Chest pain [*n* (%)]	6 (29)	NA	NA
PROMs at visit 1			
Fatigue Severity Scale	43 (22–49)	23.5 (17–32)	0.03
Dyspnoea‐12	6 (1–3)	0 (0–0))	<0.001
MoCA	26 (25–28)	29 (27–30)	0.07
PHQ‐9	5 (1–12)	2 (0–4)	0.06
Blood biomarkers at visit 1			
CRP (mg/L)	2.4 (2.1)	2.2 (3.0)	0.9
TNFα (pg/mL)	5.8 (1.7)	4.8 (1.7)	0.4
IL‐6 (pg/mL)	0.2 (13.2)	0.2 (21.9)	1.0
HbA1C (mmol/mol)	38 (3.9)	38 (3.1)	0.8
Troponin (Ug/L)	3 (1.6–3.5)	1.6 (1.6–2.9)	0.2
BNP (ng/L)	44 (16–71)	50 (31–64)	0.4
ALT (U/L)	26 (19–42)	27 (21–36)	1.0
Total bilirubin (μmol/L)	11 (10–16)	10 (7.5–14)	0.5

*Note*: The data are presented as means (standard deviation), *n* (%) or medians (inter‐quartile range).

Abbreviations: ALT, alanine transaminase; BNP, brain natriuretic peptide; CK, creatine kinase; CRP, C‐reactive protein; DEXA, dual‐energy x‐ray absorptiometry; HbA1C, glycosylated haemoglobin; IL‐6, interleukin 6; IMV, invasive mechanical ventilation; MoCA, Montreal Cognitive Assessment; NA, not applicable; NIV, non‐invasive ventilation; PHQ‐9, Personal Health Questionnaire; PROM, patient‐reported outcome measures; TNFα, tumour necrosis factor alpha.

### 3.1 Physical function, whole‐body fat‐free mass, leg muscle volume and in vivo mitochondrial function

The SPPB scores were less (*P* = 0.004) and there was a trend to lower (*P* = 0.07) average daily step count in patients compared to controls. Measures of DEXA‐derived whole‐body fat‐free mass (FFM), leg muscle volume, maximal strength and fatiguability were comparable between groups (Table 3). The iEMG demonstrated slower motor unit firing rates in patients compared to controls [β coefficient (95% confidence interval) COVID‐19 patient versus control: −0.7 (−1.4 to 0.007), *P* = 0.05].

**TABLE 3:** Comparison of habitual physical function, physical activity levels and muscle metabolic properties between patients and controls.

	**Patients**	**Controls**	***P***
SPPB	11 (10–11), *n *= 21	12 (11.25–12), *n *= 10	0.004
5 chair sit to stand (s)	12.8 (11.1–14.0), *n *= 21	10.3 (9.6–11.1), *n *= 10	0.02
Average daily step count (steps/day)	3626 (2385–6337), *n *= 21	7670 (5111–10,074), *n *= 10	0.07
Fat free mass and leg muscle volume			
BSA adjusted DEXA‐derived whole body fat free mass (kg/m^2^)	27.9 (2.3), *n *= 21	26.4 (3.3), *n *= 10	0.2
BSA adjusted MRI‐derived leg muscle volume (cm^3^/m^2^)	2547 (323), *n* = 19	2384 (289), *n *= 10	0.2
Maximal voluntary strength			
DEXA‐derived lean appendicular mass adjusted QMVC (N/kg)	21.8 (4.1), *n *= 21	21.1 (4.5), *n *= 10	0.7
Fatiguability			
% decline in average work done during first 5 reps to the last 5 reps	25 (6), *n *= 18	21 (10), *n *= 10	0.1
Post‐exercise phosphocreatine recovery			
PCr recovery (*Q*_max_ ADP) (mM/s)	0.35 (0.27–0.55), *n* = 15	0.35 (0.25–0.49), *n *= 8	0.71
PCr recovery (VPCr) (mM/s)	0.31 (0.21–0.46), *n *= 15	0.27 (0.19–0.36), *n *= 8	0.53

*Note*: The data are presented as means (standard deviation) or medians (inter‐quartile range), with the number (*n*) of participants for each measure provided. Abbreviations: BSA, body surface area; MVC, maximal voluntary contraction; PCr, phosphocreatine; *Q*_max_ ADP, maximum rate of mitochondrial ATP production based on adenosine diphosphate model; SPPB, short physical performance battery test; VPCr, initial resynthesis rate of phosphocreatine.

During the ^31^P MRS protocol, both groups completed a similar number of plantar flexion contractions, and the PCr depletion at the end of the exercise was not significantly different between groups. There was no evidence that the rate of muscle PCr re‐synthesis during recovery from exercise was slower in patients, suggestive of reduced mitochondrial flux and/or mass (Table 3).

### 3.2 Metabolic health

**FIGURE 3:**
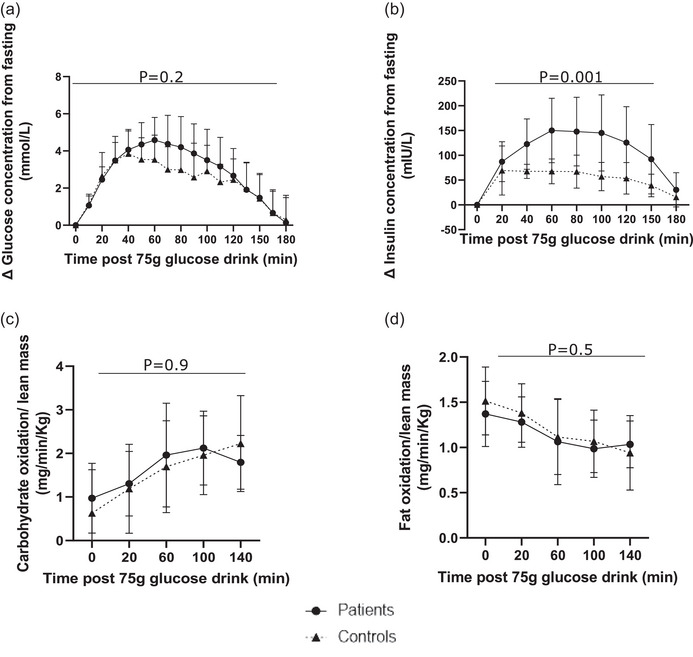
Changes in blood glucose and serum insulin concentrations, and absolute fuel oxidation rates before and in response to an oral glucose tolerance challenge. Graphs depict a comparison of change in blood glucose concentration from the fasted state (a), change in serum insulin concentration from the fasted state (b), lean mass adjusted carbohydrate oxidation rate (c), and lean mass adjusted fat oxidation rate (d) in patients (●) versus controls (▲) before and during the oral glucose tolerance test. The data are presented as means ± standard deviation. *P*‐values represent analysis from mixed effects two‐way ANOVA for patients versus controls. (a, b) Patients *n* = 21, controls *n* = 10; (c, d) patients *n* = 19, controls *n* = 10.

Changes in serum insulin and blood glucose concentrations and absolute whole‐body fuel oxidation rates in response to the OGTT are presented in Figure 3 (raw data in Supporting information Tables S1–S4). The iAUC for glucose was not different between patients [499.3 (372.6–590.0) mmol/min/L] and controls [446.1 (348.3–492.9) mmol/min/L, *P* = 0.3]. In contrast, patients exhibited a considerably greater serum insulin response to the glucose challenge [iAUC insulin patients vs. controls: 18,289 (12,497–27,448) vs. 8655 (7948–11,040) mIU/min/L, *P *< 0.001]. As a result, calculated whole‐body insulin sensitivity, Matsuda Index, was less in patients [1.68 (1.20–2.77)] compared to controls [4.90 (3.95–6.15), *P *< 0.001]. This remained significant [β coefficient Matsuda Index (95% CI): −2.21 (−3.90 to −0.52), *P* = 0.01] when adjusted for BMI. Metabolic flexibility, reflected by the change in carbohydrate and fat oxidation rates during the OGTT, was not different between groups (Figure 3c,d). HbA1C was elevated to a pre‐diabetic state in five (24%) patients and two (20%) controls (*P* = 1.0). The Matsuda Index was still different in the group with normal HbA1C [patients vs. controls, median (IQR): 1.55 (1.20–2.64) vs. 5.08 (3.43–6.47), *P* = 0.004].

DEXA‐derived whole‐body FF in all as well as those who also completed the MRI visit were comparable between groups (Table 1). Similarly, MRI‐derived calf and thigh FF, liver FF and MRS quantified VL IMCL/EMCL ratio were no different between patients and controls. Liver volume was 28% greater in patients than controls. Similarly, the FF adjusted liver *T*_1_, a measure of inflammation, was also raised in patients (Table 4).

**TABLE 4:** Comparison of MRI and MRS measures of body fat distribution between patients and controls.

	Patients	Controls	*P*
Leg FF			
Thigh FF (%)	5.3 (4.5–7.1), *n *= 19	4.6 (3.8–5.9), *n *= 10	0.3
Calf FF (%)	8.0 (6.5–11.5), *n *= 19	5.1 (4.0–12.3), *n *= 10	0.2
Thigh [IMCL] and [EMCL]			
[IMCL CH_2_] (mmol/kg)	5.1 (4.1–8.5), *n *= 16	7.0 (4.8–13.7), *n *= 10	0.7
[EMCL CH_2_] (mmol/kg)	16.0 (10.5–28), *n *= 16	18.2 (9.7–30.9), *n *= 10	0.8
IMCL:EMCL	0.45 (0.28), *n *= 16	0.67 (0.42), *n *= 10	0.1
Liver adiposity and inflammation			
BSA adjusted liver volume (cm^3^/m^2^)	783 (120), *n *= 19	612 (100), *n *= 10	<0.001
Fat fraction (%)	9.0 (4.0–14.0), *n *= 19	3.9 (3.6–7.2), *n *= 10	0.13
* T*_2_* (%)	21.2 (19.8–23.2), *n *= 19	20.8 (20–22.1), *n *= 10	0.60
* T*_1_ corrected for FF (ms)	879 (874–1057), *n *= 17	830 (824–869), *n *= 7	0.04

*Note*: The data are presented as means (standard deviation) or medians (inter‐quartile range), with the number (*n*) of participants for each measure provided. Abbreviations: EMCL, extra‐myocellular lipid; FF, fat fraction; IMCL, intra‐myocellular lipid; *T*_2_*: *T*_2_ star.

### 3.3 Cardiovascular function

**FIGURE 4:**
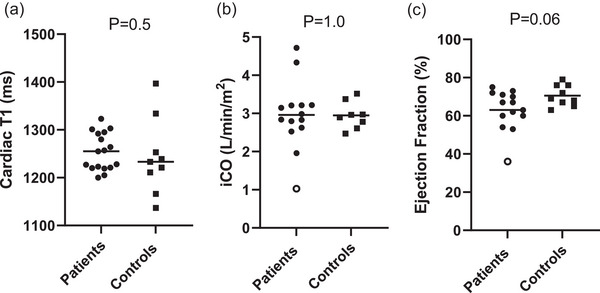
Indices of ventricular inflammation and function in the resting state. Graphs depict (a) cardiac *T*_1_, which provides a measure of the degree of myocardial inflammation/fibrosis; (b) cardiac index (iCO); and (c) left ventricular ejection fraction. Data are individual and median (a) and mean (b, c) values for patients (●) and controls (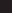). *P*‐values are derived from the Mann–Whitney *U*‐test for (a) and Student's *t*‐test for (b, c). The data point (○) in the patient group represents the individual who suffered an acute cardiac event during acute COVID‐19 infection. (a) Patients *n* = 17, controls *n* = 9; (b, c) patients *n* = 14, controls *n* = 8. iCO, cardiac output indexed for body surface area.

At rest, myocardial *T*_1_ and iCO were not statistically different in patients compared to controls (Figure 4a,b); there was a slightly lower left ventricular ejection fraction (EF) in patients (*P* = 0.06), though almost all had normal values (Figure 4c). One patient with a clinically abnormal left ventricular systolic function (EF 36%), suffered an acute myocardial infarction during their hospital stay with acute COVID‐19.

**FIGURE 5:**
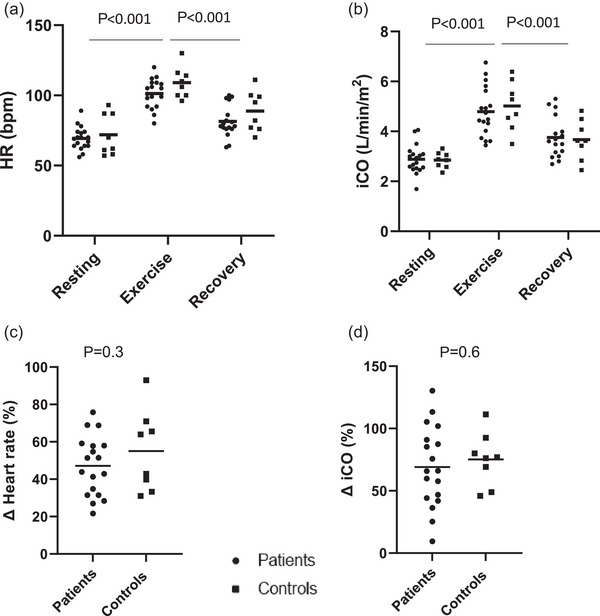
Cardiac response to steady‐state supine exercise and recovery. Data are individual and mean values for patients (●) and controls (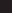). (a) and (b) The heart rate and cardiac index, respectively, at rest, during exercise and recovery. (c) and (d) The change in heart rate and cardiac index, respectively, from rest to steady‐state exercise. *P*‐values are derived from two‐way mixed effects ANOVA for (a, b), with volunteer status (rest, exercise, recovery) as the main effect. *P*‐values relate to Student's *t*‐test (patients versus controls) for (c, d). Patients *n* = 18, controls *n* = 8. BPM, beats per minute; HR, heart rate; iCO, cardiac output indexed for body surface area.

Exercise induced a significant, expected increment in steady‐state exercise HR and iCO from rest in all participants (Figure 5a,b). These increases were no different between groups (Figure 5c,d), as was the same for exercise workload [power patients: 56 (50–72) vs. controls: 64 (60–72) W, *P* = 0.3].

### 3.4 Brain structure, volume and function

There was no evidence of global atrophic changes in patients [GMV/TIV: 41 (39–43)%; cortical thickness: 2.5 (0.1) mm] compared to controls [GMV/TIV: 41 (36–47)%, *P* = 1.0; cortical thickness: 2.5 (0.2) mm, *P* = 0.6].

**FIGURE 6:**
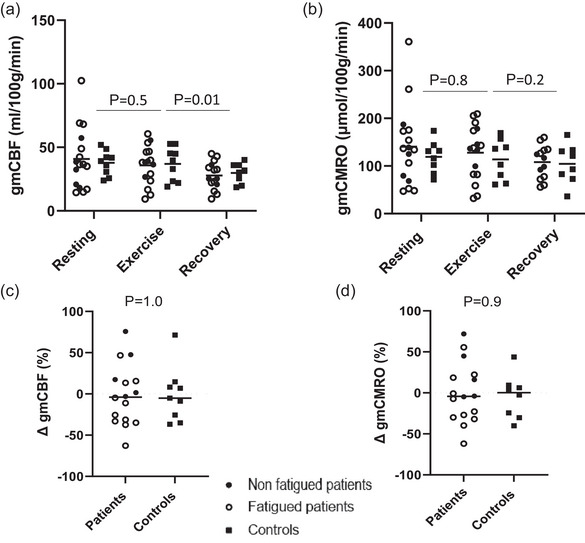
Cerebral haemodynamic response to steady‐state supine exercise and recovery. Data are individual and mean (a, b) or median (c, d) values for non‐fatigued patients (●), fatigued patients (○) and controls (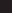). (a, b) Grey matter corrected cerebral blood flow (gmCBF) (a), and grey matter corrected cerebral metabolic rate of oxygen (gmCMRO) (b) at rest, during exercise and recovery. (c, d) gmCBF (c) and gmCMRO (d) percentage from rest to exercise. *P*‐values are derived from two‐way mixed effects ANOVA for (a, b) with volunteer status (rest, exercise recovery) as the main effect. *P*‐values relate to the Mann–Whitney *U*‐test for (c, d). (a, c) Patients *n* = 17, controls *n* = 9; (b, d) patients *n* = 17, controls *n* = 8.

Steady‐state exercise did not induce a significant change in gmCBF and gmCMRO across all subjects from rest (Figure 6a,b). As such, the percentage change in gmCBF and gmCMRO in response to exercise did not significantly differ between groups (Figure 6c,d).

### 3.5 Comparison of muscle properties and insulin sensitivity in patients with and without perceived fatigue

Patients with greater perceived fatigue had worse SPPB scores and reduced average daily physical activity compared to those without perceived fatigue (Table 5), despite no differences in whole body FFM, leg muscle volume, quadriceps strength, objective fatiguability, cardiac function or GM volume.

**TABLE 5:** Comparison of muscle composition, physical functionality, habitual function, physical activity levels, insulin sensitivity, cardiac and brain health in patients with and without perceived fatigue.

	Perceived fatigued	No perceived fatigued	*P*
Average daily step count	2520 (1999–3621), *n* = 12	6337 (4603–7930), *n* = 9	0.01
SPPB	10 (9–10.5), *n* = 12	11 (11–12), *n* = 9	0.004
Muscle properties			
Quadriceps MVC/lean appendicular mass (N/kg)	21.1 (4.4), *n* = 12	22.8 (3.8), *n* = 9	0.4
BSA adjusted total lean mass (kg/m^2^)	27.9 (2.9), *n* = 12	28.1 (1.4), *n* = 9	0.8
BSA adjusted lower limb muscle volume (cm^3^/m^2^)	2569 (318), *n* = 11	2560 (295), *n* = 8	0.9
Δ in average work done during first 5 reps and last 5 reps (%)	27 (6), *n* = 10	23 (6), *n* = 8	0.2
Insulin sensitivity			
Matsuda Index	1.3 (1.1–2.3), *n* = 12	2.5 (1.7–3.6), *n* = 9	0.08
Cardiac function			
* T*_1_ (ms)	1268 (42), *n* = 9	1239 (31), *n* = 8	0.1
Resting iCO (L/min/m^2^)	2.8 (0.6), *n* = 10	3.0 (0.5), *n* = 8	0.9
Δ in iCO from rest to exercise (%)	61% (35), *n* = 10	79% (28), *n* = 8	0.2
Resting HR (BPM)	71 (12), *n* = 10	71 (8), *n* = 8	1.0
Δ in HR from rest to exercise (%)	42% (16), *n* = 10	52% (16), *n* = 8	0.2
Cerebral properties			
Grey matter volume/TIV (%)	41 (37–43), *n* = 11	42 (40–44), *n* = 5	0.4
Resting gmCBF (mL/100 g/min)	44.1 (27.0), *n* = 11	33.4 (15.7), *n* = 5	0.7
Δ in gmCBF from rest to exercise (%)	−25 (−34.9 to 13.4), *n* = 11	18 (−0.76 to 61.6), *n* = 5	0.02
Resting gmCMRO (μmol/100 g/min)	150 (95), *n* = 11	120 (48), *n* = 5	0.8
Δ in gmCMRO from rest to exercise (%)	−23 (−31.9 to 18.6), *n* = 11	16 (−4.3 to 58.4), *n* = 5	0.09

*Note*: The data are presented as means (standard deviation) or medians (inter‐quartile range). Abbreviations: BPM, beats per minute; BSA, body surface area; gmCBF, grey matter corrected cerebral blood flow; gmCMRO, grey matter and CBF corrected cerebral metabolic rate of oxygen; HR, heart rate; iCO, cardiac output adjusted for BSA; MVC, maximal voluntary contraction; SPPB, short physical batter performance.

Patients with perceived fatigue had a slightly reduced (*P* = 0.08) whole‐body insulin sensitivity (Table 5). There was a significant difference in %Δ gmCBF from rest to exercise in fatigued patients compared to non‐fatigued. This difference remained significant [β coefficient %Δ gmCBF (95% CI): −57 (−108 to −6), *P* = 0.03] when adjusted for percentage change in iCO with exercise.

The hospital stay and inpatient treatment did not impact outcome measures in patients; there were no differences in the Matsuda Index, FSS and HbA1C between patients who did and did not receive dexamethasone during the acute illness. Further, these variables were not associated with the length of stay (Tables 6 and 7).

**TABLE 6:** Comparison of insulin sensitivity (Matsuda Index), HbA1C, fatigue severity scale and SPPB physical function between patients who did and did not receive dexamethasone during their acute COVID‐19‐related hospital stay.

	Received dexamethasone (*n* = 14)	Did not receive dexamethasone (*n* = 7)	*P*
Matsuda Index	1.50 (1.20–2.67)	2.55 (1.13–3.86)	0.36
HbA1C (mmol/mol)	37 (34–42)	38 (37–42)	0.40
Fatigue Severity Scale	40 (21–49)	46 (29–62)	0.45
SPPB	11 (10–12)	10 (9–11)	0.62

*Note*: The data are presented as medians (inter‐quartile range). Abbreviations: HBA1C, glycosylated haemoglobin; SPPB, short physical performance battery test.

**TABLE 7:** Correlation (*R* ^2^) for inpatient length of stay versus insulin sensitivity (Matsuda Index), HbA1C, fatigue severity scale and SPPB physical function in patients during their acute COVID‐19‐related hospital stay.

	*R* ^2^	*P*
Matsuda Index	0.0087	0.69
HbA1C mmol/mol	0.05	0.33
Fatigue severity scale	0.035	0.41
SPPB	0.37	0.003

Abbreviations: HBA1C, glycosylated haemoglobin; LOS, length of stay; SPPB, short physical performance battery test.

## 4 DISCUSSION

Through a comprehensive whole‐body metabolic and physiological assessment, we report greater insulin resistance assessed by OGTT in adults post‐severe COVID‐19 compared to controls, though whole‐body blood glucose control and fuel oxidation rates were comparable. Patients also experienced a greater perception of fatigue and breathlessness, as well as demonstrating worse functional mobility, through worse SPPB scores. Importantly, there were consistently no differences in robust measures of muscle mass, strength, fatiguability, neuromuscular and muscle mitochondrial function, and cardiac and cerebral structure and function at rest or during exercise.

The greater insulin resistance is in line with previous literature (Montefusco et al., 2021), although historically, this has relied on isolated fasting glucose and insulin concentrations rather than robustly exploring glycaemic control. The more comprehensive assessment of metabolic health conducted here revealed a novel observation that a clearly more overt post‐prandial hyperinsulinaemic response in patients maintained glycaemic control and metabolic flexibility comparable to control volunteers. The degree of hyperinsulinaemia was similar to previous literature reporting post‐glucose challenge insulin iAUC in adults with previously known impaired glucose tolerance test (RISE Consortium, 2018). Further, the reduced insulin sensitivity was present even in patients with a normal HbA1C compared to controls, suggesting relatively acute alterations in glucose disposal before it can be clinically detected. The insulin resistance could not be obviously explained by greater whole‐body, liver or leg adiposity or IMCL content, in the patient group, all of which are recognised drivers of insulin resistance and metabolic inflexibility (Goodpaster & Sparks, 2017; Ye, 2013). Whilst the BMI was marginally greater in patients than controls, this is a poor surrogate for body composition, with DEXA‐ and MRI‐derived measures being more robust. However, even when adjusted for BMI, insulin resistance remained greater. Alternative mechanisms are, therefore, likely to have contributed to the increased insulin resistance, as discussed.

It is recognised that viral‐induced pro‐inflammatory states promote insulin resistance. Šestan et al. (2018) infected mice with influenza A and cytomegalovirus to demonstrate that viral‐induced interferon‐γ (IFN‐γ) downregulated the insulin receptor in muscles and promoted compensatory hyperinsulinaemia. The role of such cytokines in mediating a pro‐inflammatory state has also been highlighted following COVID‐19 (Mehandru & Merad, 2022). Interestingly, there were no significant differences in serum cytokine concentrations between patients and controls in the current study. In keeping with this, glucose control, metabolic flexibility and muscle mass, all targets for cytokine‐mediated dysregulation (Crossland et al., 2019), were no different between patient and control groups.

The majority of the patients were subject to prolonged bed rest during their acute hospitalisation. Physical inactivity is strongly associated with poor metabolic health and increased mortality (Haskell et al., 2009). Bed‐rest and reduced step count‐related studies in healthy individuals have demonstrated that even short periods of immobilisation (Shur et al., 2022) or reduced ambulatory activity (Krogh‐Madsen et al., 2010) can result in sizeable sustained reductions in whole‐body glucose uptake. A possible mechanism stems from a reduction in insulin‐stimulated glucose disposal, seen predominantly at a peripheral muscle level (Stuart et al., 1988). Certainly, patients are more likely to exhibit a sedentary behaviour if they experience persistent fatigue. Here, we reported that the average daily step count was less for patients than controls (though not statistically significant; *P* = 0.07), and moreover, those with greater perceived fatigue were more sedentary and exhibited a greater insulin resistance than non‐fatigued patients, which indicates possible insulin resistance at a peripheral muscle level. A recent study by McAuley et al. (2023) identified physical inactivity as a driver for limited recovery and frailty in individuals 1 year after hospital discharge from COVID‐19, which we can integrate into a plausible mechanism. This has important implications, as encouraging physical activity in such individuals may be appropriate to improve metabolic health.

There is an abundance of evidence highlighting the persistent burden of symptomatology experienced by people after an acute SARS‐CoV2 infection (Evans et al., 2021). Whilst prolonged recovery following hospitalisation is to be expected, the burden of persistent symptoms in individuals post‐COVID‐19 appears greater than in patients discharged following community‐acquired pneumonia (Pick et al., 2019). The prevalence of fatigue experienced by COVID‐19 patients in this study is comparable to reports from Evans et al. (2021). Larger BSA‐corrected liver volume and degree of hepatic inflammation (indicated by liver *T*_1_) in patients observed here have also been reported previously and may indicate alterations in liver health (Raman et al., 2021). The worse physical function exhibited by COVID‐19 patients in the SPPB test, particularly those that experienced a greater perception of fatigue, is also an important finding as it highlights the consequences of symptoms on patients’ everyday functionality. The methodologies used in the study are the most comprehensive measures to date, utilising a robust and multifaceted approach to phenotyping muscle properties and stressing the body with low‐level exercise to assess a wider profile of cardiac and cerebral physiology. Therefore, the lack of perturbation in muscle strength, mass, fatiguability and metabolism, as well as heart and brain structure and function suggest that an as yet unknown alternative process may be responsible for the greater perception of fatigue and worse physical function. It should be noted that patients demonstrated slightly reduced resting EF compared to controls. However, this is unlikely to explain the greater perception of fatigue, given that the values were not clinically abnormal (Petersen et al., 2017) for the majority of patients. Other studies reporting deficits in muscle strength, evidence of myocardial inflammation and regional GM volume loss in patients recovering after a severe SARS‐CoV2 infection, were mainly conducted 3–4 months after the acute infection (Dennis et al., 2021; Raman et al., 2021; Tanrıverdi et al., 2021).

The sub‐group analysis comparing patients with and without fatigue revealed significant differences in the CBF response to exercise. Such observations have previously been observed in healthy, sedentary young and older individuals using a similar exercise‐based protocol (Hale, 2018). However, the difference was present only between patient sub‐groups and not patients and controls. Nevertheless, the results here may indicate alterations in cerebral neuronal activity as a cause for perceived fatigue in patients. Reduced CBF in non‐hospitalised individuals experiencing post‐COVID fatigue appears to be prominent in the orbitofrontal regions of the brain, where the motor cortex is located (Kim et al., 2022). Therefore, alterations in this region may cause blunting of neuronal drive to skeletal muscle fibres particularly during activity which would manifest as premature cessation of exercise, a ‘central’ cause for fatigue (Taylor et al., 2016). Reports of altered regional CBF have also been documented in other post‐viral fatigue syndromes (Shan et al., 2020). The slower firing rates at normalised contraction levels in patients observed during the iEMG protocol also suggest possible altered central drive, but this is not supported by deficits in muscle strength or fatiguability. So, whilst reduced CBF could possibly have influenced the perception of effort and functional mobility, it cannot have been a significant factor in determining functional capacity.

The comprehensive assessment of different organs, using multiple robust modalities, is a strength of this study. Whilst other researchers have utilised static MRI measures to study whole‐body pathology, to our knowledge this is the first study to employ exercise and glucose challenges to assess metabolic and physiological homoeostasis under dynamic and well‐controlled experimental conditions. A further strength of this study is that it overcomes the limitations of previous work by the inclusion of a control group of similar demography to the patients recruited into the study.

We recruited patients following severe COVID‐19, as we expected any abnormalities to be most apparent in this cohort. However, the cohort encompassed individuals who required invasive or non‐invasive ventilation as well as those on high‐flow oxygen therapy only. Moreover, the recruitment of patients spanning the first and subsequent infection waves meant that not all received dexamethasone, as some would have been treated prior to the outcomes of the RECOVERY trial (RECOVERY Collaborative Group, 2021). Sub‐group analysis between patients, who underwent a hospital admission, with and without perceived fatigue enabled further delineation of pathologies potentially associated with the experience of persistent fatigue.

There are some methodological considerations. The cross‐sectional nature of the study and the lack of pre‐COVID‐19 data on metabolic health make it difficult to delineate whether patients developed insulin resistance following the infection or whether these were pre‐existing features that made patients more susceptible to severe COVID‐19 (Cariou et al., 2020). However, an extensive review of medical records from admission gave no indication of a pre‐COVID diagnosis of diabetes in recruited patients. Further, the comparator control group had a similar metabolic profile. The results are also in keeping with previous literature assessing the longitudinal effects of COVID, which suggests insulin resistance in patients persists months after the initial infection (Montefusco et al., 2021). Although this is a small observational study, the planned sample size for patients versus controls for several parameters was met based on the historical information we had for these outcomes. A retrospective power calculation for the other core parameters was conducted based on detecting a physiologically relevant difference for iAUC insulin, IMCL and Matsuda Index, if present, and for these outcomes as well we calculated >80% power.

The control group comprised healthy individuals rather than patients hospitalised with other conditions, which prevents ascertaining if the findings are potentially due to COVID‐19 or hospitalisation in general. However, the degree of persisting symptoms after COVID‐19 is striking in comparison to patients discharged due to other pathologies. This suggests that the mechanisms contributing to post‐COVID debilitation may extend beyond the impact of hospitalisation. It should be further noted that no association between the length of inpatient stay and outcomes of insulin sensitivity were observed in this study. Moreover, including a control group of hospitalised individuals would have resulted in greater heterogeneity by comparing one disease pathology with a range of other disease states that may have led to admission. Recruiting healthy individuals with a similar demography to the patients allowed appropriate identification of pathology or maladaptation.

We have identified a group of individuals who experience a greater perception of fatigue, with evidence of reduced functional mobility and in whom there is a greater degree of insulin resistance. Importantly, this perturbation in metabolic function is not detected by simple clinical blood tests such as HbA1C and may, therefore, warrant closer observation. Further, addressing step count to maximise glucose disposal may be appropriate in some patients with perceived fatigue.

There is a pressing need to aid recovery back to full health in individuals experiencing persistent post‐COVID‐19 symptoms. This whole‐body observational study provides novel insight and informs forward strategy for rehabilitation interventions in a sub‐group of individuals, without previous known morbidity, who are now limited in their activities of daily living following severe COVID‐19.

## AUTHOR CONTRIBUTIONS

Charlotte E. Bolton, Paul L. Greenhaff, Susan T. Francis, Ayushman Gupta, Tricia M. McKeever, James Bonnington, Ian Hall and Mathew Piasecki contributed to the conceptualisation of the study design. Charlotte E. Bolton, Ian P. Hall, Janet M. Lord, Rachael A. Evans and Christopher E. Brightling established funding acquisition. Susan T. Francis, Christopher R. Bradley and Eleanor F. Cox were responsible for developing the MR‐based scan sequences. Charlotte E. Bolton, Ayushman Gupta and James Bonnington invited participants. Ayushman Gupta conducted the literature search and led the study conduct, visits and data collection along with Rosemary Nicholas, Jordan J. McGing, Aline V. Nixon, Joanne E. Mallinson and Christopher R. Bradley. Ayushman Gupta, Jordan J. McGing, Rosemary Nicholas, Joanne E. Mallinson, Aline V. Nixon, Mathew Piasecki, Eleanor F. Cox and Charlotte E. Bolton contributed to the various aspects of data analysis, with Ayushman Gupta having access to and verifying all the data. Charlotte E. Bolton, Paul L. Greenhaff, Susan T. Francis, Mathew Piasecki, Ian P. Hall, Jordan J. McGing, Tricia M. McKeever, Rosemary Nicholas and Ayushman Gupta were involved in reviewing and interpreting the data. Ayushman Gupta drafted the initial manuscript and was responsible for collating comments from co‐authors and subsequent revision. Charlotte E. Bolton, Paul L. Greenhaff and Susan T. Francis supervised Ayushman Gupta in PhD and project and were responsible for the decision to submit the manuscript along with Ayushman Gupta. All authors contributed to the critical review and revision of the manuscript. All authors have read and approved the final version of this manuscript and agree to be accountable for all aspects of the work in ensuring that questions related to the accuracy or integrity of any part of the work are appropriately investigated and resolved. All persons designated as authors qualify for authorship, and all those who qualify for authorship are listed.

## CONFLICT OF INTEREST

Outside of the work submitted, C.E.Bo. reports grants from UKRI P‐HOSP COVID, NUH Trust and charitable donation from the University of Nottingham. C.E.Bo. is also a member of the iDMC but in an unrelated disease – COPD. S.T.F. reports funding from UKRI and NIHR grants for work not directly related to this project. C.E.Br. received funding from NIHR and MRC for research outside this manuscript and reports grants and consulting fees from GlaxoSmithKline, AstraZeneca, Sanofi, Regeneron, Roche, Genentech, Boehringer Ingelheim, Novartis, Chiesi, 4Dpharma, Mologic and Areteia. I.P.H. reports funding from UKRI and NIHR in the form of grant and Senior Investigator Award for work not directly related to this project. I.P.H. is also a Vice Chair and Trustee for Asthma and Lung UK and receives speaker fees from Boehrineger‐Ingelheim. R.A.E. reports funding and grants from UKRI/MRC and NIHR/Wolfson Foundation, respectively. Further, R.A.E. received consulting fees from AstraZeneca, speaker fees from Boehringer Ingelheim for a lecture on Long COVID, support from Chiesi to attend BTS meeting virtually and is also a member of the ERS group for pulmonary rehabilitation. All other authors declare no conflicts of interest.

## Supporting information

Table S1. Individual participant data for blood glucose response during the oral glucose tolerance test. DYNxxx represent patients and DYNxxxc represent controls.

Table S2. Individual participant data for serum insulin response during the oral glucose tolerance test. DYNxxx represent patients and DYNxxxc represent controls.

Table S3. Individual participant carbohydrate oxidation rates/lean mass during the oral glucose tolerance test. DYNxxx represent patients and DYNxxxc represent controls. CHO: carbohydrate oxidation

Table S4. Individual participant fat oxidation rates/lean mass during the oral glucose tolerance test. DYNxxx represent patients and DYNxxxc represent control

## Data Availability

The protocol is available online at https://www.nrru.org/post/dynamic‐assessment‐of‐multi‐organ‐level‐dysfunction‐in‐patients‐recovering‐from‐covid‐19‐dynamo. Data are available upon reasonable request from the corresponding author.
