# Supplementary material for: DYNamic Assessment of Multi‐Organ level dysfunction in patients recovering from COVID‐19: DYNAMO COVID‐19
Source: Exp Physiol. 2024 Jun 24;109(8):1274–91. doi: 10.1113/EP091590 (PMC11291868; doi:10.1113/EP091590)
Supplement: Supplementary file 1 — Table S1. Individual participant data for blood glucose response during the oral glucose tolerance test. DYNxxx represent patients and DYNxxxc represent controls. [file EPH-109-1274-s003.docx]

**Supplementary results**

| **Change in blood glucose from fasting (mmol/L)** | **Minutes after oral glucose challenge** | | | | | | | | | | | | | | | |
| --- | --- | --- | --- | --- | --- | --- | --- | --- | --- | --- | --- | --- | --- | --- | --- | --- |
| **Participant** | **10** | **20** | **30** | **40** | **50** | **60** | **70** | **80** | **90** | **100** | **110** | **120** | **135** | **150** | **165** | **180** |
| DYN001 | 1.28 | 2.73 | 3.58 | 3.92 | 4.53 | 5.25 | 5.35 | 5.45 | 4.95 | 4.57 | 3.72 | 3.01 | 2.68 | 1.96 | 0.85 | -0.01 |
| DYN002 | 1.13 | 2.22 | 3.19 | 3.86 | 3.83 | 3.67 | 3.62 | 3.75 | 3.70 | 3.37 | 3.18 | 2.49 | 2.07 | 0.72 | -0.23 | -1.32 |
| DYN003 | 0.54 | 1.44 | 2.26 | 3.14 | 3.80 | 4.73 | 5.02 | 5.24 | 4.87 | 4.57 | 3.99 | 3.60 | 3.04 | 2.61 | 2.07 | 1.85 |
| DYN004 | 1.39 | 2.82 | 4.09 | 4.62 | 4.73 | 4.84 | 5.11 | 4.59 | 3.49 |  | 2.65 | 2.14 | 1.45 | 1.22 | 0.87 | 0.72 |
| DYN005 | 1.35 | 3.11 | 4.78 | 5.65 | 6.15 | 6.95 | 7.45 | 7.25 | 6.95 | 6.25 | 6.05 | 5.45 | 4.65 | 3.12 | 2.00 | 0.99 |
| DYN006 | 0.42 | 1.49 | 1.61 | 2.13 | 1.78 | 1.98 | 1.72 | 1.82 | 1.45 | 1.22 | 1.29 | 0.99 | 0.85 | 1.00 | -0.02 | -1.04 |
| DYN007 | 1.12 | 2.48 | 3.64 | 4.39 | 4.03 | 4.08 | 3.76 | 2.92 | 3.33 | 1.24 | 0.87 | 0.82 | 0.04 | 0.31 | -0.13 | -0.63 |
| DYN008 | 2.88 | 4.14 | 4.27 | 3.95 | 4.70 | 4.61 | 4.09 | 2.48 |  | 1.48 | 1.00 | 0.73 | -0.75 |  |  |  |
| DYN009 | 0.70 | 2.08 | 3.51 | 4.48 | 4.74 | 5.38 | 5.73 | 5.87 | 5.43 | 4.71 | 4.74 | 4.14 | 3.23 | 1.76 | 0.55 | -0.75 |
| DYN010 | 1.19 | 3.18 | 4.16 | 4.69 | 4.91 | 5.11 | 4.65 | 4.61 | 4.10 | 3.44 | 2.39 | 1.91 | 0.21 | -1.19 | -1.94 | -2.27 |
| DYN011 | 1.07 | 2.49 | 2.84 | 4.22 | 4.28 | 4.59 | 4.39 | 3.70 | 2.20 | 1.78 | 1.75 | 0.52 | -0.41 | -1.00 | -1.24 | -1.11 |
| DYN012 | 0.76 | 3.31 | 4.70 | 5.25 | 6.25 | 6.35 | 6.35 | 6.35 | 5.25 | 5.75 | 5.65 | 4.90 | 3.55 | 3.59 | 2.51 | 1.14 |
| DYN013 | 0.98 | 1.55 |  | 3.28 | 4.35 | 4.27 | 2.86 | 2.60 | 2.86 | 3.23 | 3.51 | 2.72 | 2.74 | 2.83 | 1.06 |  |
| DYN014 | 1.45 | 3.31 | 4.70 | 5.56 | 5.76 | 6.46 | 6.66 | 6.66 | 5.96 | 5.56 | 5.66 | 5.06 | 3.32 | 2.05 | 1.14 | 0.10 |
| DYN015 | 0.70 | 2.15 | 2.99 | 3.55 |  | 3.71 | 3.62 | 3.56 | 2.65 | 3.29 | 2.98 | 2.31 |  | 1.51 | 0.05 | 0.08 |
| DYN016 | 1.02 | 2.68 | 5.21 | 5.81 | 5.91 | 5.91 | 5.31 | 4.81 | 4.70 | 4.88 | 4.46 | 4.50 | 2.98 | 1.56 | 1.22 | 0.34 |
| DYN017 | 0.70 | 1.97 | 2.86 | 3.81 | 4.44 | 4.79 | 4.89 | 5.36 | 5.03 | 4.39 | 3.85 | 2.77 | 2.54 | 1.48 | 0.80 | 0.27 |
| DYN020 | 0.65 | 2.15 | 3.05 | 3.90 | 3.57 | 3.08 | 1.65 | 1.62 | 1.16 | 0.71 | 1.02 | 1.42 | 0.91 | 0.29 | -0.24 | -0.43 |
| DYN028 | 1.04 | 1.92 | 2.51 | 3.31 | 3.43 | 3.50 | 3.11 | 2.48 | 1.75 | 2.08 | 2.21 | 2.45 | 2.01 | 1.22 | 0.50 | 0.45 |
| DYN030 | 1.37 | 2.12 | 2.22 | 2.09 | 2.13 | 3.36 | 4.29 | 4.80 | 4.80 | 4.70 | 3.29 | 2.33 | 2.06 | 2.76 | 2.78 | 4.02 |
| DYN031 | 0.60 | 2.37 | 3.57 | 3.83 | 3.72 | 3.63 | 2.36 | 2.39 | 2.72 | 2.95 | 2.30 | 1.79 | 1.07 | 1.80 | 0.67 | 0.22 |
| DYN019c | 0.33 | 2.50 | 3.25 | 2.55 | 2.39 | 2.98 | 3.66 | 3.98 | 4.04 | 4.20 | 3.64 | 2.70 | 1.85 | 1.74 | 0.61 | 0.18 |
| DYN021c | 1.15 | 2.09 | 1.91 | 2.72 | 2.74 | 2.32 | 2.50 | 2.90 | 2.46 | 2.35 | 1.76 | 1.76 |  | 1.21 | -0.17 | -1.03 |
| DYN022c | 1.52 |  | 3.27 | 4.25 |  | 4.51 | 3.92 | 4.25 | 3.62 | 3.43 |  | 2.75 | 1.43 | 1.47 | 0.86 | 0.66 |
| DYN023c | 1.47 | 3.65 | 2.47 | 1.48 | 1.30 | 0.58 | 0.39 | 0.76 | 0.27 | 3.51 | 3.01 | 2.30 | 3.34 | 3.22 | 1.41 | 0.30 |
| DYN025c | 1.66 | 2.59 | 3.98 | 4.29 | 4.54 | 4.96 |  |  | 5.36 | 5.36 |  | 4.91 | 3.88 | 1.93 | 0.08 | -1.09 |
| DYN027c | 0.39 | 0.73 | 1.90 | 3.19 | 3.79 | 4.25 | 3.94 | 4.15 | 4.03 | 3.27 | 3.49 | 2.96 | 2.45 | 2.35 | 2.74 | 2.34 |
| DYN029c | 1.86 | 5.35 | 6.21 | 5.67 | 4.81 | 4.65 | 4.08 | 3.17 | 2.10 | 1.89 | 1.25 | 0.48 | -1.15 | -1.83 | -1.72 | -1.31 |
| DYN032c | 1.24 | 2.64 | 3.60 | 4.47 | 3.90 | 3.24 | 2.88 | 2.52 | 2.32 | 2.28 | 1.88 | 2.65 | 2.38 | 2.36 | 2.16 | 1.80 |
| DYN033c | 1.18 | 2.33 | 3.99 | 4.78 | 4.64 | 4.15 |  | 3.42 | 2.42 | 2.58 | 2.31 | 1.81 | 1.32 | 0.67 | 0.16 | -0.64 |
| DYN034c | 0.68 | 2.05 | 4.40 | 5.08 | 3.73 | 3.66 | 2.63 | 1.67 | -0.85 | 0.22 | 1.27 | 2.13 | 2.12 | 1.22 | 0.47 | 1.69 |

**Table S1. Individual participant data for blood glucose response during the oral glucose tolerance test.** DYNxxx represent patients and DYNxxxc represent controls**.**
