# Supplementary material for: DYNamic Assessment of Multi‐Organ level dysfunction in patients recovering from COVID‐19: DYNAMO COVID‐19
Source: Exp Physiol. 2024 Jun 24;109(8):1274–91. doi: 10.1113/EP091590 (PMC11291868; doi:10.1113/EP091590)
Supplement: Supplementary file 2 — Table S2. Individual participant data for serum insulin response during the oral glucose tolerance test. DYNxxx represent patients and DYNxxxc represent controls. [file EPH-109-1274-s001.docx]

**Supplementary results**

| **Change in serum insulin concentration from fasting (mIU/L)** | **Minutes after oral glucose challenge** | | | | | | | |
| --- | --- | --- | --- | --- | --- | --- | --- | --- |
| **Participant** | **20** | **40** | **60** | **80** | **100** | **120** | **150** | **180** |
| DYN001 | 114.8 | 148.02 | 221.82 | 286.8 | 287.28 | 185.01 | 150.39 | 68.02 |
| DYN002 | 59.74 | 84.04 | 69.91 | 66.27 | 62.06 | 62.22 | 22.4 | 6.63 |
| DYN003 | 70.31 |  | 207.57 | 221.48 | 258.33 | 212.99 | 173.95 | 84.18 |
| DYN004 | 68.85 | 68.71 | 107.52 | 97.43 |  | 57.95 | 54.3 | -1.3 |
| DYN005 | 87.07 | 125.68 | 189.46 | 269.53 | 219.79 | 189.03 | 126.4 | 73.3 |
| DYN006 | 21.42 | 32.76 | 39.88 | 35.66 | 35.11 | 26.25 | 30.66 | 1.70 |
| DYN007 | 66.89 | 124.89 | 172.82 | 133.78 | 71.88 | 50.12 | 37.65 | 13.43 |
| DYN008 | 131.87 | 130.15 | 160.26 | 95.69 | 100.86 | 42.4 |  |  |
| DYN009 | 42.72 | 103.84 | 162.94 | 174.88 | 125.45 | 209.45 | 111.27 | 33.85 |
| DYN010 | 44.97 | 73.94 | 99.17 | 110.38 | 128.35 | 106.88 | 18.85 | 8.24 |
| DYN011 | 86.58 | 123.1 | 137.55 | 134.3 | 76.73 | 33.89 | 1.7 | -7.94 |
| DYN012 | 87.06 | 84.91 | 90.18 | 120.65 | 125.20 | 141.08 | 164.95 | 61.94 |
| DYN013 | 107.02 | 207.26 | 313.01 | 155.22 | 200.54 | 216.43 | 242.86 |  |
| DYN014 | 87.14 | 141.8 | 174.45 | 192.67 | 216.62 | 225.45 | 169.74 | 86.75 |
| DYN015 | 184.08 | 223.02 | 218.48 | 239.58 | 237.58 | 230.07 | 150.47 | 41.23 |
| DYN016 | 41.58 | 128.01 | 89.98 | 107.99 | 149.44 | 176.82 | 105.28 | 60.78 |
| DYN017 | 69.47 | 91.58 | 124.33 | 159.52 | 144.98 | 120.55 | 42.72 | 10.82 |
| DYN020 | 163.56 | 226.24 | 224.67 | 141.51 | 43.11 | 74.42 | 3.18 | -29.19 |
| DYN028 | 73.63 | 121.65 | 111.25 | 90.14 | 92.04 | 103.15 | 49.57 | 22.39 |
| DYN030 | 127.99 | 136.71 | 154.06 | 218.46 | 236.91 | 135.87 | 143.45 | 44.68 |
| DYN031 | 94.29 | 77.31 | 85.71 | 55.19 | 88.58 | 35.68 | 41.41 | -2.25 |
| DYN019c | 72.70 | 59.38 | 81.59 | 111.86 | 114.29 | 94.73 | 72.7 | 7.25 |
| DYN021c | 45.2 | 50.36 | 50 | 60.81 | 53.12 | 37.55 | 25.88 | -0.49 |
| DYN022c | 53.84 | 71.28 | 92.55 | 83.97 | 81.06 | 72.93 | 45.12 | 13.96 |
| DYN023c | 181.19 | 54.54 | 11.58 | 12.22 | 69.18 | 25.67 | 36.45 | 8.45 |
| DYN025c | 43.96 | 62.10 | 69.01 | 123.17 |  | 110.32 | 71.95 | 8.49 |
| DYN027c | 21.73 | 52.72 | 59.46 | 77.6 | 58.84 | 37.32 | 34.86 | 37.45 |
| DYN029c | 133.42 | 91.36 | 79.17 | 59.62 | 20.65 | 4.43 | 2.86 | 6.63 |
| DYN032c | 52.52 | 77.89 | 72.66 | 47.1 | 39.18 | 50.13 | 45.70 | 30.86 |
| DYN033c | 35.28 | 71.19 | 60.15 | 54.06 | 49.3 | 44.25 | 9.96 | 1.16 |
| DYN034c | 53.44 | 85.67 | 100.19 | 40.11 | 29.35 | 59.15 | 45.76 | 41.82 |

**Table S2. Individual participant data for serum insulin response during the oral glucose tolerance test.** DYNxxx represent patients and DYNxxxc represent controls**.**
