# Supplementary material for: DYNamic Assessment of Multi‐Organ level dysfunction in patients recovering from COVID‐19: DYNAMO COVID‐19
Source: Exp Physiol. 2024 Jun 24;109(8):1274–91. doi: 10.1113/EP091590 (PMC11291868; doi:10.1113/EP091590)
Supplement: Supplementary file 3 — Table S3. Individual participant carbohydrate oxidation rates/lean mass during the oral glucose tolerance test. DYNxxx represent patients and DYNxxxc represent controls. CHO: carbohydrate oxidation [file EPH-109-1274-s002.docx]

**Supplementary results**

| **CHO/lean mass (mg/min/kg)** | **Minutes after oral glucose challenge** | | | | |
| --- | --- | --- | --- | --- | --- |
| **Participant** | **0** | **20** | **60** | **100** | **140** |
| DYN003 | -0.09 | 0.59 | 1.04 | 1.71 | 1.59 |
| DYN004 | 0.93 | 0.74 | 1.41 | 1.42 | 0.66 |
| DYN005 | 1.60 | 0.66 | 1.96 | 2.17 | 1.61 |
| DYN006 | 1.12 | 1.72 | 3.05 | 3.14 | 2.99 |
| DYN007 | 0.92 | 1.10 | 1.07 | 2.01 | 1.57 |
| DYN008 | 1.49 | 2.62 | 2.42 | 1.95 | 1.75 |
| DYN009 | -0.18 | -0.88 | -0.60 | 0.92 | 0.73 |
| DYN010 | 0.78 | 1.68 | 2.23 | 3.08 | 2.74 |
| DYN011 | 2.47 | 1.73 | 2.64 | 3.03 | 2.42 |
| DYN012 | 1.73 | 1.73 | 1.73 | 1.72 | 1.68 |
| DYN013 | 0.51 | 2.02 | 3.04 | 1.98 | 2.03 |
| DYN014 | 1.38 | 0.95 | 1.83 | 2.59 | 2.37 |
| DYN015 | 0.39 | 1.39 | 1.91 | 1.80 | 1.29 |
| DYN016 | 0.89 | 1.29 | 2.22 | 1.64 | 1.91 |
| DYN017 | 1.67 | 1.52 | 2.43 | 2.16 | 1.64 |
| DYN020 | 0.34 | 1.82 | 5.22 | 4.38 | 2.25 |
| DYN028 | 1.40 | 0.94 | 0.71 | 0.93 | 1.16 |
| DYN030 | 1.85 | 1.89 | 2.19 | 2.42 | 2.25 |
| DYN031 | -0.75 | 1.27 | 0.79 | 1.29 | 1.48 |
| DYN019c | 1.67 | 1.74 | 2.54 | 2.80 | 3.24 |
| DYN021c | 1.16 | 1.86 | 0.58 | 2.65 | 3.46 |
| DYN022c | 1.47 | 1.88 | 2.75 | 2.33 | 1.74 |
| DYN023c | -0.63 | -0.07 | 0.42 | 0.09 | 2.63 |
| DYN025c | -0.03 | 1.18 |  | 3.01 | 3.00 |
| DYN027c | 1.40 | 1.29 | 1.94 | 1.61 | 1.34 |
| DYN029c | 1.40 | 2.94 | 3.18 | 2.57 | 3.41 |
| DYN032c | 1.03 | 0.44 | 2.05 | 1.36 | 0.94 |
| DYN033c | -0.09 | 1.17 | 1.85 | 1.20 | 2.12 |
| DYN034c | -1.10 | -0.53 | 1.64 | 1.97 | 0.36 |

**Table S3. Individual participant carbohydrate oxidation rates/lean mass during the oral glucose tolerance test.** DYNxxx represent patients and DYNxxxc represent controls**.** CHO: carbohydrate oxidation
