# Supplementary material for: DYNamic Assessment of Multi‐Organ level dysfunction in patients recovering from COVID‐19: DYNAMO COVID‐19
Source: Exp Physiol. 2024 Jun 24;109(8):1274–91. doi: 10.1113/EP091590 (PMC11291868; doi:10.1113/EP091590)
Supplement: Supplementary file 4 — Table S4. Individual participant fat oxidation rates/lean mass during the oral glucose tolerance test. DYNxxx represent patients and DYNxxxc represent control [file EPH-109-1274-s004.docx]

**Supplementary results**

| **Fat oxidation/ lean mass (mg/min/kg)** | **Minutes after oral glucose challenge** | | | | |
| --- | --- | --- | --- | --- | --- |
| **Participant** | **0** | **20** | **60** | **100** | **140** |
| DYN003 | 1.54 | 1.23 | 1.06 | 0.77 | 0.86 |
| DYN004 | 1.13 | 1.37 | 1.02 | 0.97 | 1.28 |
| DYN005 | 0.99 | 1.38 | 0.89 | 0.89 | 0.81 |
| DYN006 | 1.31 | 0.98 | 0.46 | 0.56 | 0.60 |
| DYN007 | 0.97 | 1.11 | 1.34 | 0.67 | 0.87 |
| DYN008 | 1.30 | 0.86 | 1.21 | 1.10 | 1.19 |
| DYN009 | 1.59 | 2.06 | 2.23 | 1.44 | 1.51 |
| DYN010 | 1.77 | 1.16 | 0.95 | 0.75 | 0.68 |
| DYN011 | 0.82 | 1.18 | 0.85 | 0.64 | 0.70 |
| DYN012 | 1.18 | 1.18 | 1.12 | 1.11 | 1.07 |
| DYN013 | 1.34 | 0.90 | 0.43 | 0.88 | 0.84 |
| DYN014 | 1.26 | 1.43 | 1.29 | 0.92 | 0.94 |
| DYN015 | 1.88 | 1.47 | 1.12 | 1.23 | 1.30 |
| DYN016 | 1.27 | 1.21 | 0.81 | 1.06 | 0.89 |
| DYN017 | 1.07 | 1.22 | 0.90 | 0.93 | 1.09 |
| DYN020 | 1.94 | 1.63 | 0.10 | 0.54 | 1.33 |
| DYN028 | 1.21 | 1.58 | 1.65 | 1.55 | 1.33 |
| DYN030 | 1.30 | 1.22 | 1.17 | 1.04 | 1.14 |
| DYN031 | 2.18 | 1.17 | 1.63 | 1.67 | 1.23 |
| DYN019c | 1.13 | 1.21 | 0.69 | 0.61 | 0.53 |
| DYN021c | 1.33 | 1.18 | 2.08 | 0.78 | 0.53 |
| DYN022c | 1.25 | 1.04 | 0.79 | 0.90 | 1.16 |
| DYN023c | 1.61 | 1.76 | 1.42 | 1.64 | 0.43 |
| DYN025c | 2.01 | 1.48 |  | 0.91 | 0.83 |
| DYN027c | 1.00 | 1.14 | 0.97 | 1.05 | 1.22 |
| DYN029c | 1.39 | 0.95 | 1.00 | 1.15 | 0.70 |
| DYN032c | 1.49 | 1.69 | 1.07 | 1.46 | 1.61 |
| DYN033c | 1.78 | 1.47 | 1.09 | 1.43 | 0.93 |
| DYN034c | 2.15 | 1.89 | 0.93 | 0.74 | 1.47 |

**Table S4. Individual participant fat oxidation rates/lean mass during the oral glucose tolerance test.** DYNxxx represent patients and DYNxxxc represent control
